# H-coil repetitive transcranial magnetic stimulation does not improve executive function in patients with chronic peripheral neuropathic pain: a randomized sham-controlled crossover study

**DOI:** 10.3389/fpsyt.2024.1401008

**Published:** 2024-07-17

**Authors:** Nadine Farnes, Henrik B. Jacobsen, Audun Stubhaug, Sara M. Vambheim

**Affiliations:** ^1^ Department of Pain Management and Research, Division of Emergencies and Critical Care, Oslo University Hospital, Oslo, Norway; ^2^ Institute of Clinical Medicine, University of Oslo, Oslo, Norway; ^3^ Mind Body Lab, Department of Psychology, University of Oslo, Oslo, Norway; ^4^ Department of Research and Development, Division of Emergencies and Critical Care Oslo University Hospital, Oslo, Norway

**Keywords:** rTMS (repetitive transcranial magnetic stimulation), executive functions, peripheral neuropathic pain, chronic pain, neuromodulation

## Abstract

**Introduction:**

Deep rTMS is an increasingly popular noninvasive brain stimulation technique which has shown promise for treating cognitive impairments. However, few studies have investigated the cognitive effects it could exert in patients with chronic peripheral neuropathic pain. Therefore, we aimed to assess the effects of deep rTMS on executive functioning in patients with peripheral neuropathic pain, in a randomized, double-blind crossover trial.

**Methods:**

In total, 17 patients were randomly assigned to receive both active and sham deep H-coil rTMS targeting the primary motor cortex. Each treatment period consisted of five daily rTMS sessions. Selected tests of executive functioning from the CANTAB test battery (paired associates learning, stop signal task, spatial working memory and multitasking test) were performed at baseline, and at 1 week and 3 weeks follow-ups.

**Results:**

We did not find any significant interactions between time and treatment for the measures of executive functioning for the patient group, or for patients with reduced cognition compared to normative means.

**Conclusion:**

High-frequency deep H-coil rTMS targeting the hand area of the primary motor cortex and delivered over 5 consecutive days did not improve executive functioning in patients with chronic peripheral neuropathic pain.

**Clinical trial registration:**

https://clinicaltrials.gov/, identifier NCT05488808.

## 1 Introduction

Chronic neuropathic pain has a range of negative impacts on patients’ health and wellbeing (1–3). This includes poorer cognitive functioning compared to patients with mixed neuropathic and nociceptive pain (4). Moreover, a significant proportion of neuropathic pain patients perform below normal levels on tests of executive functioning, such as psychomotor speed, attention, working memory, verbal learning, and general intelligence (5). Patients with peripheral neuropathic pain appear to have specific impairments in resource-demanding visual encoding and retrieval compared to fibromyalgia patients and healthy individuals (6). Impairments in executive functioning are detrimental to daily functioning and are rated by patients with chronic pain as one of the more bothersome symptoms (7). Together, these findings underscore the importance of focusing on executive functioning in the assessment and treatment of patients with chronic pain (8).

In recent years, repetitive transcranial magnetic stimulation (rTMS) has gained recognition as a safe and promising treatment option for neuropsychiatric disorders, as well as for pain relief in chronic neuropathic pain (9). High frequency rTMS (>5Hz) targeting the dorsolateral prefrontal cortex is given to relieve symptoms of depression (10). On the other hand, a primary motor cortex (M1) target, most often targeting the hand motor cortical area disregarding the painful site, is given to relieve chronic pain (9, 11–13). However, there is some debate as to whether this strategy or somatotopic matching of M1 to the painful region is most optimal for pain relief.

Aside from its effect on psychological symptoms and pain, rTMS delivered with the standard figure-8 coil and targeting the prefrontal cortices has shown to improve cognitive function in patients with major depression (14). In fact, figure-8 coil rTMS to the M1 was given to fibromyalgia patients with chronic pain, resulting in improvement in cognitive performance on tests of attention and inhibition in the active rTMS group, although the difference when compared to the sham group was not significant (15).

The figure-8 coil is designed to target focal, superficial areas of the cortex. Newer designs, such as the H-coil, can reach deeper into the brain and affect larger brain volumes, termed deep rTMS. Indeed, H-coil rTMS to the prefrontal cortices has shown to improve cognitive functions in Alzheimer’s patients (16). The extensive reach of the H-coil rTMS has the potential to influence not only the hand motor cortex, but also neighboring cortical regions such as the dorsolateral prefrontal cortex, of which rTMS stimulation has been effective in improving cognitive functioning (14, 17, 18). This may offer dual benefits to patients suffering from chronic neuropathic pain, particularly those who also face impairments in executive functions. To date, only one study has investigated the effects of high frequency H-coil rTMS in chronic neuropathic pain patients on executive functions (19). Deep rTMS was delivered to the anterior cingulate cortex with an H-coil and to the insular cortex with a double-cone coil in patients with central neuropathic pain. A slight improvement in verbal fluency was seen after rTMS treatment compared to sham, however this effect did not persist when correcting for multiple analyses.

The lack of improved cognitive function in chronic pain patients after rTMS may be attributed to brain area target or measures of cognitive functioning. Measures of cognitive functions that have shown to be impaired in the specific patient populations should be used when the goal is to improve performance after rTMS treatment. Thus, the effect of rTMS on executive functioning in patients with neuropathic pain should be further examined.

A prominent theory of cognitive impairments in chronic pain hypothesizes that pain experiences and intensity could be disruptive for executive functioning and attention, potentially driving cognitive impairments (20). Thus, pain-relieving treatments, such as deep rTMS, may restore impairments in executive functions seen in peripheral neuropathic pain (6). No studies have yet explored the effects of M1-rTMS to the motor cortex on executive functioning in peripheral neuropathic pain patients. We therefore investigated the effects of 5 daily sessions of M1 H-coil rTMS on executive functioning in patients with chronic peripheral neuropathic pain. Specifically, we wanted to examine whether rTMS could improve executive functioning in this patient group. We also assessed whether sex, age, pain duration, and spontaneous pain at baseline could predict the effect of rTMS on executive functioning.

## 2 Methods

This study was a part of a project investigating analgesic efficacy of deep H-coil rTMS to the hand area of the motor cortex in peripheral neuropathic patients. Here, we present secondary analyses from this project, focusing on the effects of rTMS on executive functioning. The protocol was approved by the local ethics committee, Regional Committees for Medical and Health Research Ethics (REK/428116) and pre-registered in clinicaltrials.gov: NCT05488808. All patients provided written informed consent before inclusion in the study and conformed the Declaration of Helsinki and Good Clinical Practice Guidelines.

### 2.1 Participants

Patients with peripheral neuropathic pain were recruited to and treated at the Department of Pain Management and Research at Oslo University Hospital from August 2022 to August 2023. Eligible subjects were between 18 and 80 years old and diagnosed with peripheral neuropathic pain fulfilling the criteria of probable or definite neuropathic pain (21). They had to experience daily pain with a numerical rating scale (NRS) intensity of at least 4/10 that had lasted for minimum 3 months, and usual pain intensity ≥ 4 of 10 over the past 24 hours using the numerical rating scale of the Brief Pain Inventory (22). Stable pharmacological treatment or no pharmacological treatment for pain at least 1 month prior to inclusion was required and had to remain unchanged throughout the study period. Subjects could not have phantom limb pain after limb amputation, any clinically significant or unstable medical or psychiatric disorder, be protected by law (guardianship or tutelage measure), have previous or current substance abuse, pending litigation, or contraindications to rTMS. Contraindications to rTMS included past severe head trauma, history of or ongoing epilepsy or cerebral tumor, past neurosurgical intervention, intracranial hypertension, implanted devices such as cardiac pacemaker and neurostimulator, cochlear implants, pregnancy, or lactation (women of childbearing age were required to have negative pregnancy test at inclusion and to be using contraception). Other exclusion criteria were more severe pain conditions than peripheral neuropathic pain, inability to understand the study protocol and fill out the forms, and participation in other ongoing research protocol or recent past protocol the last month prior to inclusion.

### 2.2 Experimental design

Participants were randomly assigned to receive five consecutive days of either active or sham rTMS to the hand area of the M1. After a washout period of nine weeks, they were assigned to receive five days of either active or sham rTMS, in line with a counterbalanced crossover design. Thus, each participant received active and sham rTMS treatment. Altogether, each treatment period included five treatment days (D1-D5), and two follow-up visits at one (W1) and three weeks (W3) after treatment completion.

Randomization lists were generated by a computer which produced block randomization with varying block sizes to secure allocation blinding. The study was conducted double-blinded, with both patients and investigators being unaware of treatment group allocation. This was ensured by personalized magnetic cards inserted into the rTMS stimulator, which automatically selected the delivery of active or sham treatment.

Deep rTMS was administered with the Brainsway H7-coil (Brainsway, Jerusalem, Israel) connected to a Magstim Rapid2 stimulator (Magstim, Whitland, UK) that delivered repetitive stimulation. The stimulation target was the primary motor cortex of the hand corresponding to the hand on the painful side. In cases of bilateral pain, the left hemisphere was targeted, as done in previous rTMS studies in chronic pain (9, 23, 24). Stimulation target was reproduced for each treatment session based on markings in a cap worn by the participant, recording the spatial coordinates. Each treatment session consisted of 30 series of 10 Hz pulses with intertrain intervals of 20 seconds, resulting in 3000 total pulses administered at 80% of resting motor threshold (RMT). The RMT was defined as the lowest intensity that elicited a motor response of the first dorsal interosseous muscle of at least 50 µV peak-to-peak in at least five out of 10 successive trials (25), and was determined before the first rTMS session. RMT was determined using the Cadwell EMG Serra Summit system (Cadwell, Washington, USA). The sham coil was encapsulated inside the active coil and produced a negligible electric field inside the brain, with a similar acoustic artefact as the active coil (26).

Patients wore earplugs to minimize adverse effects of hearing and were seated in a reclining chair with a vacuum pillow to stabilize the head during stimulation.

### 2.3 Measures of executive function

Executive functioning was recorded at baseline, one week (W1) and three weeks (W3) after the last stimulation sessions, and was assessed by selected computerized, non-verbal tests from the self-administrated neuropsychological CANTAB battery (27).

We used the following measures from the test battery: Paired Associates Learning (PAL), Stop Signal Task (SST), Spatial Working Memory (SWM) and Multitasking Test (MTT).

PAL assessed attention-demanding cued recall, measuring encoding and retrieval. Participants were presented with boxes that are “opened” in a random order, containing, or not containing a pattern inside. The participant had to remember which box contains which patterns. We used the extended PAL version to mitigate ceiling effects on the most difficult stages. We used two outcomes: the number of errors made on the last, most difficult stage (PALTEA8), where maximum number of errors are 32, and number of errors adjusted for estimated number of errors in stages they did not teach (PALTEA28), where maximum number of errors were 70.

SST is a test of stop signal response inhibition and measured the executive component of inhibitory control. The tests consisted of five blocks of 64 trials each, where participants were presented to an arrow on the screen and had to respond or withhold response in the direction of the arrow dependent on an audio tone. The outcome variable was the estimate of the stop signal reaction time (SSRT) in milliseconds, measuring the speed of the inhibitory process (28). Maximum signal reaction time was 1000 milliseconds. Lower values signified higher performance.

SWM measures the executive component of updating, assessing the ability to retain and manipulate visuospatial information (27). Through the process of elimination, participants found which boxes on the screen that contained a yellow token and had to remember the placements in order to find successive tokens. The extended SWM version was used to mitigate ceiling effects on the most difficult stages. We used the two outcomes SWMS and SWMBE8. SWMS measured the strategy of avoiding errors, referring to the frequency with which the participant began a search in a new box during the most challenging stage. Minimum strategy value was two and maximum strategy value was 14. SWMBE8 measured the number of errors when revisiting a box where a token previously is found across eight token trials. Maximum errors were 74. A lower score indicated better performance on both measures.

MTT measured executive function by the ability to handle conflicting information. Participants were shown an arrow on the left or right side of the screen, pointing in either direction. A cue indicated a rule to select according to the placement of the arrow or pointing direction of the arrow. The rule changed within a single task. The outcome was the number of trials with incorrect responses, where the maximum was 160.

In addition to individual scores, we calculated a composite score of executive functioning. The composite score was the averaged combined z-scores of the PAL (PALTEA28), SST, SWM (SWMBE8) and MTT. The z-score was calculated as the difference between the patient’s score and the mean score of PAL, SST, SWM or MTT divided by the standard deviation of these scores for all patients at each time point (29). A lower composite score indicated higher performance in executive functioning.

### 2.4 Patient reported outcomes

Baseline spontaneous pain was measured from one week before each treatment session (baseline) and up to three weeks after each session end. Participants rated their usual pain intensity over the past 24 hours in a diary at the same hour (end of the day) on an 11-point NRS. The average of seven days was then used as baseline measure, one week and three week follow up measures. A score of 0 indicated no pain, while 10 indicated worst pain intensity imaginable.

Baseline sleep, anxiety and depression, pain catastrophizing and functioning were measured to examine relationships with baseline executive functioning.

The insomnia Severity Index (ISI) was used to assess sleep difficulties and entail five items. The items describe subjective symptoms and consequences of insomnia (30). Higher scores suggest higher severity of insomnia.

The Hospital Anxiety and Depression Scale (HADS) was used to assess anxiety and depressive symptoms (31). Comprising 14 items, seven items measure depression and seven measure anxiety. Participants rate their responses on a 4-point Likert scale. Higher scores indicate higher likelihood of depression and/or anxiety.

The Patients’ Specific Functional Scale (PSFS) assessed everyday functioning (32). patients choose five activities of functions that they rate on an NRS, where 0 indicate inability to perform activity/function and 10 indicate ability to perform activity/function.

Pain Catastrophizing Scale was used to assess negative thoughts related to pain (33, 34). The scale has 13 items that measure negative thoughts and feelings that may occur in reaction to pain. Responses are rated on a 5-point Likert scale, and higher score suggest higher occurrence of pain catastrophizing.

### 2.5 Statistical analyses

Since this was a part of a study investigating analgesic efficacy of deep H-coil rTMS in peripheral neuropathic patients, sample size was calculated based on pain intensity. A two-sided t-test with significance level of 5% and power of 80% was used to detect a 2-point difference (using the NRS scale from zero to 10) with a common standard deviation of differences of one for the two periods. In total, 16 participants were required for this analysis.

The statistical analyses included the intention-to-treat population (ITT): all participants who were randomized to at least one stimulation session. We investigated relationships between executive functioning at baseline with age, sex, pain intensity, pain duration, anxiety and depression, sleep, pain catastrophizing and functioning using Pearson’s correlation for continuous variables and point biserial correlation for categorical variables. Pearson’s correlation was also used to investigate associations between mean change in pain intensity and mean change on the cognitive tests from baseline. We used T-tests to examine differences at baseline, with Bonferroni correction for multiple comparisons. Also, standard scores available from the CANTAB test battery for PALTEA28 and SWMS were used to divide the at sample three groups: higher cognitive functioning, defined as higher than or equal to one SD above normative values at baseline; lower executive functioning, defined as lower than or equal to one SD below normative values at baseline; and normal cognitive functioning, defined as between one SD above and one SD below normative values at baseline. The standard scores are matched for age, sex, and educational level. Changes in composite and individual scores of executive functioning from baseline were analyzed using restricted maximum likelihood estimation in a linear mixed-effects model (LMM). A significance level of p < 0.05 was considered statistically significant in all analyses.

The statistical analyses were performed using R, version 4.2.3 (35), and the linear mixed models were calculated with the *lme4* package (v1.1-34). The statistical analyses are openly accessible (https://osf.io/3xrgc/?view_only=8d843e848def4d7097d54897674d79bb).

## 3 Results

### 3.1 Baseline characteristics

In total 17 patients (58 ± 13.2 years old, 7 female) were included in the study and randomized to the order groups. Of these, one patient discontinued before the second treatment cycle, and one patient discontinued during the second treatment cycle. Therefore, 15 patients successfully completed all stimulation sessions and follow-up visits. Baseline characteristics are presented in **Table 1**.

**Table 1:** Baseline demographic and clinical characteristics of the patients.

	All patients (N = 17)
Age, mean (SD), years	58.0 (13.2)
Pain duration, mean (SD), years	6.6 (4.8)
DN4, mean (SD)	6.0 (1.1)
Sex,	
Female	7 (41.2)
Pain condition	
Radiculopathy Polyneuropathy Peripheral nerve injury Postherpetic neuralgia	4 (23.5) 6 (35.3) 6 (35.3) 1 (5.9)
Concomitant analgesic treatment^b^ Antidepressants Anticonvulsants Opioids Other^c^	12 (70.6) 6 (35.3) 9 (52.9) 5 (29.4) 7 (41.2)
Maximal pain area	
Lower limbs Upper limbs Face	11 (64.7)2 (11.8)4 (23.5)

Results are expressed as mean (SD) unless otherwise indicated.

b One or more concurrent analgesic treatments.

c Other pharmacological pain treatments, e.g., Paracetamol. Abbreviations: DN4: douleur neuropathique 4 questions.

There were no significant differences in age, sex, concomitant medication, pain condition, pain duration, usual pain intensity, sleep, functioning, anxiety and depression, pain catastrophizing or executive functioning at baseline between patients who received active before sham rTMS, or sham before active rTMS (**Table 2**). No significant differences in any of the executive functioning measures were observed between patients using (n =12) and not using (n = 5) concomitant medication for pain at baseline. However, using anticonvulsants (with no concomitant antidepressant or opioid use, n=9) was associated with greater impairment in SWM (SWMBE8) compared to those who did not use anticonvulsants (n = 8, t (10.4) = -3.4, p = 0.04, **Table 3**).

**Table 2:** Baseline differences between receiving active/sham or sham/active rTMS.

	Active/Sham (N=10)	Sham/Active (N=7)
Sex, female, N (%)	3 (30.0)	4 (57.1)
Age	60.3 (10.1)	54.8 (17.0)
Pain duration	6.9 (5.9)	6.1 (2.9)
Usual pain intensity	6.6 (1.0)	6.8 (1.2)
Multitasking Test – MTTTIC	18.5 (21.2)	15.5 (14.2)
Paired Associates Learning		
PALTEA8	19.5 (9.1)	18.5 (9.8)
PALTEA28	30.2 (14.1)	31.8 (22.6)
Stop Signal Task – SSTSSRT	253.7 (46.5)	261.3 (57.5)
Spatial Working Memory		
SWMS	8.8 (2.9)	7.5 (3.6)
SWMBE8	9.1 (6.1)	12.2 (8.9)
Composite score	-0.05 (0.4)	0.08 (0.9)
Anxiety and depression – HADS	11.4 (7.9)	10.4 (3.8)
Sleep difficulties – ISI	12.9 (9.1)	15.5 (6.9)
Pain catastrophizing – PCS	24.5 (11.5)	21.7 (10.8)
Functioning – PSFS	23.2 (12.8)	22.7 (10.6)

Results are expressed as mean (SD) unless otherwise indicated. Valid percentages displayed (missing data excluded). None of the baseline variables were significantly different between patients receiving active then sham or sham then active rTMS (p < 0.05). Lower values indicate higher performance for all measures. HADS, Hospital Anxiety and Depression Scale; PCS, Pain Catastrophizing Scale; ISI, Insomnia Severity Index; PSFS, Patient-Specific Functional Scale.

**Table 3:** Baseline measures of executive functioning divided by concomitant medication and anticonvulsive medication.

	Concomitant medication	No concomitant medication	Anticonvulsive medication	No anticonvulsive medication
Multitasking test – MTTTIC	14.3 (15.5)	24.4 (23.8)	12.0 (13.6)	23.2 (21.7)
Paired Associates Learning				
PALTEA8	17.2 (8.7)	23.8 (9.4)	19.0 (8.3)	19.2 (10.6)
PALTEA28	27.6 (18.3)	38.8 (14.0)	32.0 (18.0)	29.6 (18.0)
Stop Signal Task – SSTSSRT	255.1 (49.9)	261.2 (54.8)	259.9 (51.8.)	253.5 (50.6)
Spatial working memory				
SWMS	7.8 (3.5)	6.3 (2.6)	8.1 (3.4)	8.5 (3.1)
SWMBE8	10.3 (8.2)	10.6 (5.3)	11.7 (7.4)	9.0 (7.4) *
Composite score	-0.10 (0.8)	0.24 (0.30)	0.0 (0.8)	0.00 (0.57)

Results are expressed as mean (SD). Valid percentages displayed (missing data excluded). Lower values indicate higher performance for all measures. *A significant difference was found for spatial working memory (SWMBE8) between patients using anticonvulsive medication and patients not using anticonvulsive medication (p < 0.05).

**Figure 1:**
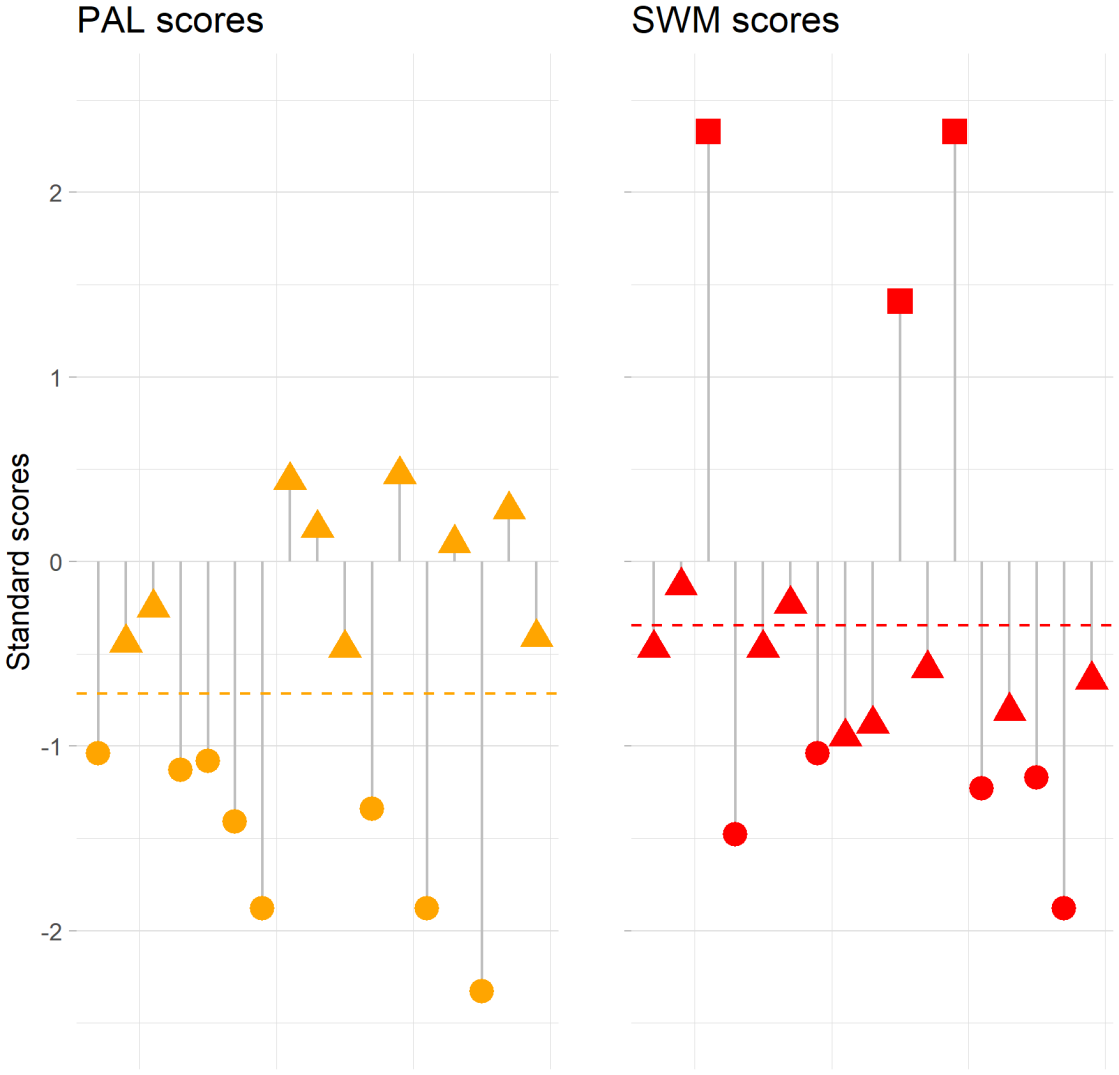
Individual standard scores for the Paired Associates Learning (PALTEA28) task and Spatial Working Memory (SWMS) task for each patient. Each lined point represents an individual patient, and the stippled line represents the mean of standard scores for each test. Patients scoring higher than or equal to one SD above the normative mean are presented as squares, patients scoring between one SD above and one SD below the normative mean are presented as triangles, and patients score lower than or equal to one SD below the normative mean are presented as circles.

We had access to normative data on two measures: PALTEA28 and SWMS. Baseline individual performance on these two measures showed that 47.1% (n=8) of the patients in our study scored one SD below the normative mean of PALTEA28, while 29.4% (n=5) scored one SD below the normative mean of SWMS (**Figure 1**).

### 3.2 Correlations between baseline cognitive and clinical measures

Multiple measures of executive function at baseline were significantly correlated with age (composite score: r = 0.79, p <0.001; PALTEA8: r = 0.63, p = 0.007; PALTEA28: r = 0.64, p = 0.005; SST: r = 0.69, p = 0.002) indicating that higher age was associated with lower performance at baseline. Being male was significantly correlated with higher baseline PAL scores (PALTEA8: r = -0.69, p = 0.001; PALTEA28: r = -0.54, p = 0.02), indicating lower baseline performance. The results also showed that symptoms of anxiety and depression were significantly correlated SST at baseline (r = 0.50, p = 0.04), indicating that higher scores of anxiety and depression was associated with lower baseline performance. Moreover, functioning was correlated MTT at baseline (r = 0.49, p = 0.04), indicating that higher functioning was associated with lower performance on this measure. Pain duration, pain intensity, sleep and pain catastrophizing were not significantly correlated with any of the executive functioning measures at baseline.

### 3.3 Effects of rTMS on executive functioning

LMM analyses showed that receiving active rTMS was significantly associated with higher overall SWM score (SWMS: t (73) = 2.53, p = 0.01), indicating lower performance. This was not significant over time, indicating that SWM scores did not change over time as a result of rTMS. No significant interactions between treatment and time for any of the outcome measures were found (**Table 4**). Age significantly predicted the response in several measures of executive function to rTMS. Higher age was significantly associated with an increase in the composite score (t (14) = 4.85, p = 0.00), PAL scores (PALTEA8:t (12) = 3.25, p = 0.01), PALTEA28: t (12) = 3.26, p =0.01), SST scores (t (13) = 2.89, p = 0.01) and SWM (t (13) = 2.25, p = 0.04) all indicative of lower performance. Sex, pain intensity and pain duration did not significantly predict response of any measures of executive functioning to rTMS.

**Table 4:** Differences in outcomes from baseline to week 1 and week 3 between patients receiving real and sham rTMS.

	Sham rTMS			Active rTMS		
	Baseline	W1	W3	Baseline	W1	W3
Usual pain intensity	6.5 (1.5)	6.5 (1.7)	6.7 (1.4)	6.5 (1.2)	6.4 (1.7)	6.2 (2.2)
Multitasking test – MTTTIC	11.8 (11.8)	10.1 (7.7)	10.1 (12.8)	16.0 (17.2)	13.5 (12.2)	12.0 (13.4)
Paired Associates Learning						
PALTEA8	17.8 (8.8)	16.6 (10.6)	17.4 (10.4)	16.8 (10.8)	16.4 (11.1)	15.9 (8.4)
PALTEA28	28.3 (17.7)	26.6 (18.4)	28.7 (19.2)	26.1 (16.1)	25.1 (17.4)	24.7 (13.5)
Stop Signal Task – SSTSSRT	238.8 (50.9)	235.5 (53.2)	227.6 (60.1)	256.2 (59.4)	233.9 (44.2)	231.2 (49.3)
Spatial working memory						
SWMS	7.2 (2.8)	6.3 (2.6)	6.6 (2.8)	8.3 (3.2)	7.6 (2.6)	6.7 (2.5)
SWMBE8	9.3 (7.5)	5.5 (6.0)	6.3 (5.9)	9.4 (6.1)	7.4 (4.6)	6.1 (5.9)
Composite score	-0.03 (0.8)	-0.06 (0.7)	0.02 (0.8)	0.03 (0.6)	0.07 (0.6)	-0.02 (0.7)

Results are expressed as mean (SD). Valid percentages displayed (missing data excluded). No significant interactions between treatment and time were found for any of the outcome measures (p < 0.05). Lower values indicate higher performance for all measures.

Subsequent analyses showed no significant interaction between treatment and time for patients who scored one SD below, at or above the normative mean of PAL (PALTEA28) or SWM (SWMS) at baseline.

We observed no correlation between mean change in pain intensity and mean change in any of the cognitive tests over the course of the study.

### 3.4 Safety

Two patients discontinued the study on the account of adverse effects. One patient experienced severe pain at stimulation site during active stimulation with nausea, fatigue, headache and pain at stimulation site, and subsequently withdrew participation. The other patient had an adverse episode immediately after sham treatment being pale, had trembling hands, light-headedness and a brief moment of unresponsiveness. ECG, pulse oximetry and blood pressure were measured and the patient were admitted for further examination. The patient had a complicated diabetic neuropathic and was further excluded from participation of the study due to further cardiac examinations at the local hospital.

## 4 Discussion

We found that 5 consecutive sessions of deep H-coil rTMS targeting the hand area of M1 did not significantly alter executive functioning in patients with chronic peripheral neuropathic pain. This is in line with previous studies on the efficacy of rTMS on executive functioning in chronic pain patients.

Two randomized controlled studies have previously investigated the effect of high frequency rTMS on executive functioning, one in chronic central neuropathic pain patients (19), and one in fibromyalgia patients (15). Like our findings, no significant effects on cognitive functioning were found after rTMS treatment in either of these studies. In the study on central neuropathic pain patients, rTMS was delivered either to the anterior cingulate cortex with a H-coil, or to the posterior insula with a double-cone coil for 5 consecutive days, followed by maintenance sessions up to 21 weeks (19). Verbal fluency showed a slight improvement after active treatment, but this effect did not remain significant when correcting for multiple analyses. Deep rTMS was also not effective in reducing pain in that study (36).

In the study on fibromyalgia patients, figure-8 coil rTMS was delivered to the hand area of the M1 for 14 stimulation sessions over 21 weeks (15). In addition to a reduction in pain intensity (37), there was an improvement in tests of attention and flexibility for active rTMS only, however the effect was not significant (15). Like the two abovementioned studies, we divided the chronic pain patient sample into higher, normal, and lower cognitive function based on normative data, finding no differences between active and sham rTMS over time for any of the groups. Nonetheless, comparisons of our findings with these studies should be done with caution as we use different outcome measures, patient populations, rTMS coils, number of rTMS session and stimulation targets.

According to the attentional model of chronic pain, pain disrupts attention processes necessary for executive functioning (20). Therefore, if pain mediates the effect of rTMS on executive functioning, the pain-relieving effects of deep rTMS could potentially resolve attentional deficits and improve executive functioning. The lack of an improvement of executive functioning in our study could be due to the pain relief being below a clinically important reduction (38). In our study, patients received five days of stimulation. Increasing the number of stimulation session may be of significance for pain relief after rTMS (9). Furthermore, we found no significant correlation between change in pain intensity and change in executive functioning from baseline, which is similar to a previous rTMS study in fibromyalgia patients (15). This could be due to the limited sample size or the minimal pain relief in our study.

To our knowledge, cognitive effects of high frequency deep H-coil rTMS to the motor cortex has not been studied previously. Most studies in psychiatric and neurological populations find improvements in cognition after rTMS have stimulated the dorsolateral prefrontal cortex (14, 17, 18). It may be that this prefrontal cortical rTMS target is necessary for cognitive improvement also for chronic neuropathic pain patients. Although we targeted the hand area of the M1, the H-coil is known to stimulate a larger brain area, reaching deeper into subcortical regions and extending to other cortical areas (39). Thus, we cannot rule out the possibility that we also stimulated prefrontal areas such as the dorsolateral prefrontal cortex. Moreover, studies reporting improvement in cognitive functioning perform rTMS for 10-15 sessions (14). Altogether, it would be interesting to see whether improvements in executive functioning in chronic neuropathic pain patients can be found in larger scale studies using similar parameters to our own, with increased number of stimulation sessions, or using a prefrontal rTMS target.

The included tests from the CANTAB battery assessed executive functioning related to visual encoding and retrieval (PAL), inhibition (SST), updating (SWM) and processing of conflicting information (MTT). Specifically, visual encoding and retrieval have shown to be impaired in patients with chronic peripheral neuropathic pain compared to healthy controls and fibromyalgia patients (6). In total, 47.1% of our sample performed one SD below the normative matched sample on this same test.

Baseline measures of executive functioning were not correlated with pain intensity, pain duration, pain catastrophizing or sleep difficulties. This contrasts a previous study investigating factors influencing cognitive functions in chronic pain patients, where higher pain intensity was associated impaired cognitive functioning in fibromyalgia patients, while pain duration found to have a U-shaped relationship with cognitive performance in neuropathic pain patients (40). The contrasting findings could be due to the limited sample size in our study. Similar to our study, sleep impairment did not affect cognitive performance in the chronic pain patients (40). Similar to previous studies on healthy individuals, we found that male gender was significantly correlated with lower baseline performance on the PAL task (41). In line with previous findings of longer inhibition reaction times on SST in individuals with depression compared to healthy controls (42), higher scores on symptoms of anxiety and depression were associated with lower baseline SST in our sample. Interestingly, higher scores on functioning were associated with lower baseline MTT. This could be due do underlying factors related to characteristics of the sample population examined, and thus warrants further investigation.

Not surprisingly, we found that older age was associated with lower performance on the composite score, PAL, and SST at baseline. Also, age predicted the overall negative performance in these measures’ response to rTMS. Executive functions tend to decline with age, likely related to changes in neural structures and functions that occur at older ages (43–45). Recognition memory have been reported to be more impaired in older patients with chronic pain compared to age-matched controls and younger patients with chronic pain (46). In addition, in older age, increased physical activity is associated with increased cognitive functioning and higher levels of pain together with decreased physical activity is associated with lower cognitive functioning (47). Although we did not investigate it, physical activity may be a mediator between old age and negative performance in executive functioning found in our study.

A limitation of our study was the small sample size. This was a secondary analysis of a study powered to examine pain intensity after rTMS treatment. For better exploration on cognitive tests in this secondary analysis, a larger group size had been advantageous. Another possible limitation was that many of the patients were treated with concomitant drugs for their neuropathic pain, which could potentially influence our findings. Although there was no difference in baseline executive functioning measures between patients who used and did not use concomitant pain treatment, patients who received anticonvulsants did perform worse on SWM (SWMBE8) at baseline compared to patients who did not receive anticonvulsants.

Thus, we cannot rule out that anticonvulsant use may have influenced our lack of finding of an improvement to rTMS on this measure. Cognitive impairments are commonly reported in chronic pain patients with trigeminal neuralgia using anticonvulsants (48). Although this patient group perform worse on cognitive measures compared to healthy controls (49), no large-scale studies have yet examined the cognitive deficits of patients with chronic peripheral neuropathic pain using anticonvulsants compared to those who do not use anticonvulsants. This should be investigated in further studies, to elucidate the role anticonvulsants play in cognitive deficits in this patient group.

Finally, executive functioning were measured earliest one week after the treatment had ended. Measuring executive functioning straight after the treatment period may inform of the immediate cognitive effects of rTMS in chronic neuropathic pain patients, although patient burden such as increased study duration should be taken into consideration.

## 5 Conclusion

Five consecutive sessions of high-frequency deep H-coil rTMS targeting the hand area of M1 did not improve executive functioning in patients with chronic peripheral neuropathic pain. Further studies with larger sample sizes are necessary to examine whether deep M1-rTMS can improve executive functioning in this patient group.

## Data availability statement

Anonymized data supporting the conclusions of this article can be made available by the authors per request.

## Ethics statement

The studies involving humans were approved by Regional Committees for Medical and Health Research Ethics (REK/428116). The studies were conducted in accordance with the local legislation and institutional requirements. The participants provided their written informed consent to participate in this study.

## Author contributions

NF: Conceptualization, Data curation, Formal analysis, Methodology, Writing – original draft. HJ: Conceptualization, Methodology, Writing – review & editing. AS: Conceptualization, Methodology, Writing – review & editing. SV: Conceptualization, Methodology, Writing – review & editing.
